# Form a League to Correct Medical Profession Abuses

**Published:** 1911-02

**Authors:** 


FORM A LEAGUE TO CORRECT MEDICAL PROFESSION ABUSES.—With the avowed object of correcting abuses in the medical profession, securing political representation and a general betterment of conditions for physicians and surgeons, the American Medico-Political Reform League has been launched in Chicago.

The league received its incorporation papers from Secretary of State Rose December 30th, and will hold its first meeting and election of officers some day this week. The incorporators, who also are the directors, are the following physicians: J. E. Waggoner, F. Tice, Lewis H. Bremerman, O. Tydings, Ralph H. Wheeler, M. G. McHugh, G. Frank Lydston, James E. Stubbs, George F. Butler, Adolph Gehrman, Charles C. O'Byrne, Henry F. Lewis and R. R. Duff.

The purpose of the league as set forth in the charter is in part as follows:

"To procure the establishment of a national bureau of health, divorced from politics; the establishment of a uniform standard of medical requirements in the several States of the Union; encouragement and co-operation with all movements and legislation for food reform which shall be fair and impartial and founded on scientific premises; encouragement of political preferment of physicians as tending to secure just representation for the profession; encouragement of measures for the correction of hospital and dispensary abuses of charity."—Chicago paper.

